# Rapid clearance of bacteria from maternal bloodstream after delivery in pregnancies complicated by preterm pre-labor rupture of the membranes

**DOI:** 10.21203/rs.3.rs-7359456/v1

**Published:** 2025-09-11

**Authors:** Catalin S. Buhimschi, Guomao Zhao, Kara M. Rood, Lindsey Solden, Shaylyn D. Webster, Manel Guessas, Stephanie Hearne, Anthony Moussa, Sonia Guessas, Hongwu Jing, Kathryn Berryman, William E. Ackerman, Irina A. Buhimschi

**Affiliations:** University of Illinois Chicago College of Medicine; University of Illinois Chicago College of Medicine; The Ohio State University College of Medicine; The Abigail Wexner Research Institute, Nationwide Children’s Hospital; The Ohio State University College of Medicine; The Abigail Wexner Research Institute, Nationwide Children’s Hospital; The Abigail Wexner Research Institute, Nationwide Children’s Hospital; The Abigail Wexner Research Institute, Nationwide Children’s Hospital; The Abigail Wexner Research Institute, Nationwide Children’s Hospital; University of Illinois Chicago College of Medicine; The Ohio State University College of Medicine; University of Illinois Chicago College of Medicine; University of Illinois Chicago College of Medicine

## Abstract

Preterm pre-labor rupture of membranes (PPROM) increases maternal and neonatal sepsis risk, yet its association with maternal microbial translocation and systemic inflammation remains unclear. We investigated whether PPROM is linked to microbial DNA in maternal blood (MB) and inflammatory responses around delivery. In 66 PPROM patients (median GA: 32±1 weeks), MB was collected pre-delivery and within 1 hour postpartum. Fetal membranes (FM) and placental tissues were sampled immediately after delivery. Bacterial load and diversity were analyzed via 16S rDNA qPCR and sequencing. Maternal cytokines were quantified by multiplex immunoassay, and fetal inflammatory exposure (Triple I) assessed using histological chorioamnionitis (HCA), cord blood haptoglobin, and IL-6. Bacterial DNA was detected in maternal blood (MB) pre- and post-delivery, with a significant postpartum decline (p=0.004). FM carried higher bacterial load and biodiversity than placenta (p<0.001), dominated by Mycoplasma spp. Maternal IL-6 and IL-10 levels rose postpartum (p<0.05), particularly in cases with fetal inflammatory exposure. Limited overlap was found between MB and tissue microbial taxa. In conclusion, bacterial DNA is detectable in maternal circulation in patients with PPROM before birth but rapidly clears postpartum alongside a robust cytokine surge, suggesting efficient clearance and dynamic inflammatory changes.

## Introduction

Fetal membranes (FM) demarcate the amniotic fluid (AF) compartment and cushion the fetus by protecting it from environmental dangers through complex mechanical, biochemical and immunological features. During gestation, FM grow and adapt mechanically and biochemically to pregnancy until parturition is initiated through mechanisms not yet fully understood.

When FM are intact and the mother is not in labor, the AF cavity is sterile in 99% of the cases. However, the emergence of more sensitive culture-independent techniques for microorganism detection challenged the notion that the intrauterine environment is truly microbe free., Heated debates have surrounded the assertion that AF, FM and the placenta harbor specific microbiomes independent of the integrity of the FM.^,,,,^ If true, these findings could be relevant for physiologic and high-risk gestations including those complicated by preterm labor and preterm pre-labor rupture of the membranes (PPROM).

Although bacterial attachment to the amnio-chorion is possible and has been well demonstrated, the mechanisms responsible for microbial seeding of the gestational sac and the fetus are less clear when FM are intact. Conversely, in the instance of PPROM, given the breach in the integrity of the FM, vaginal microbes ascend to the gestational sac, from where they may gain access to the fetus. In this clinical scenario, balancing the risk of early delivery with that of expectant management is challenging because expeditious delivery increases the risk of neonatal prematurity-related complications both, before and after 34 weeks.^,,^ If PPROM occurs < 34 weeks, in the absence of signs or symptoms of intra-amniotic infection and inflammation (Triple I), expectant management is recommended. Still, even in the absence of signs or symptoms of Triple I maternal and neonatal sepsis may silently occur.^,’^ Rarely, hematogenous spread of bacteria from endogenous reservoirs to the feto-placental unit is possible. This alternative infection dissemination pathway is supported by existing similarities between placental, oral cavity (e.g., Firmicutes, Proteobacteria), and gastro-intestinal tract (e.g., Enterococcus, Streptococcus, Escherichia-Shigella) microbiomes in various pregnancy-associated clinical scenarios.

High-throughput, culture-independent technologies can be used to interrogate the microbiome at high taxonomic resolution. Previously, we investigated the microbial communities in AF and cord blood of patients who delivered preterm in the context of Triple I by sequencing of the bacterial 16S ribosomal RNA gene., We identified *Bergeyella*, *Fusobacterium nucleatum*, and *Sneathia sanguinegens* in cord blood of premature fetuses with suspect or confirmed early-onset sepsis with 72% of bacterial taxa found in cord blood having a matching taxon in AF. While these observational studies support the notion that bacteria can be translocated from the AF into the fetal compartment, less is known about the possible translocation of bacteria, from the gestational sac into maternal blood (MB) of mothers with PPROM immediately after birth.

Infection is a well-established risk factor for and complication of PPROM. The current hypothesis takes in consideration that delivery is ensuing when the gestational sac’s inflammatory response reaches a critical point. It is thus reasonable to propose that placental, FM and AF microbiomes play a key role in triggering a maternal compartment systemic inflammatory response which leads to preterm delivery. Herein, we sought to investigate: **1)** if delivery of the fetus in pregnancies complicated by PPROM is associated with a surge of bacterial DNA and cytokines in maternal circulation and **2)** if FM and placenta harbor specific microbiomes that could be found in MB shortly after birth.

## Results

### Demographic and clinical characteristics of the subjects.

The demographic and clinical characteristics of the study population are presented in Table 1. Of the 66 enrolled mothers, 61 were pregnant with singletons and 5 were pregnant with twins. All twin pregnancies were di-chorionic di-amniotic (di-di). The median gestational age (GA) at enrollment was 32 [28–34] weeks. PPROM complicated pregnancy at <34 weeks in 68% (45/66) and at <28 weeks in 26% (17/66) of the cases. Patients with PPROM occurring at earlier GA had longer latency periods. While all patients delivered preterm, those with a late PTB (34–36^6/7^ weeks) were the patients where PPROM occurred at >34 weeks GA. These patients either progressed spontaneously or were promptly delivered per clinical protocol. Thirty-five percent (23/66) of the patients delivered by Cesarean.

Selected clinical and research laboratory characteristics for pregnancies and newborns exposed (EXP) and non-exposed (NEXP) to Triple I are displayed in Table 2. Exposure status of the newborn was determined through the combination of cord blood haptoglobin (Hp) and interleukin-6 (IL-6) using an algorithm described in the Methods section. Twin pregnancies were considered EXP if at least one of the fetuses as identified as EXP. Analysis of the 71 cord blood specimens identified 53% (35/66) of pregnancies as NEXP and 47% (31/66) as EXP to Triple I. Of the 5 twin pregnancies included in the study, 2 were classified as EXP, each with only one of the twins identified as EXP. In the remainder of the 3 twin pregnancies, both fetuses were concordantly NEXP. Patients with EXP pregnancies experienced PPROM at an earlier GA and delivered earlier compared to patients with NEXP pregnancies (p<0.001 for both). The latency period was significantly longer for patients with EXP pregnancies (p<0.001).

In pregnancies where an EXP newborn was identified, the maternal WBC count was significantly higher immediately prior to birth (p<0.001), but not at admission for PPROM (p=0.072) or post-partum (p=0.128) as determined by the clinical laboratory workup. Patients with EXP pregnancies exhibited abdominal pain more frequently (p=0.025). While the frequency of a full diagnosis of clinical chorioamnionitis did not differ between the EXP and NEXP pregnancies, placental pathological examination more frequently diagnosed histological chorioamnionitis and funisitis among EXP newborns (p<0.001 for both). Newborns identified as EXP had lower birth weights, a higher frequency of 1-min Apgar score <7, and a higher percentage of bands on the differential. No other clinical hematological abnormalities were significant except for an elevated nucleated RBC count (p<0.001).

There were marked differences in cord blood interleukin-6 (IL-6) and cord blood haptoglobin (Hp concentration among EXP and NEXP newborns (p<0.001 for both, Table 2). All NEXP newborns (40/40) had cord blood IL-6 <100 pg/mL and switch-off Hp pattern. Of EXP newborns, 28/31 (90%) had switch-on Hp pattern (EXP haptoglobinemic). Three newborns were considered EXP in the setting of cord blood IL-6 >100 pg/mL but a switch-off Hp pattern (EXP ahaptoglobinemic), as previously described.[i]^,[ii]^

### Bacterial biomass in MB before and after delivery and relationships with circulating cytokine levels.

**Figure 1:**
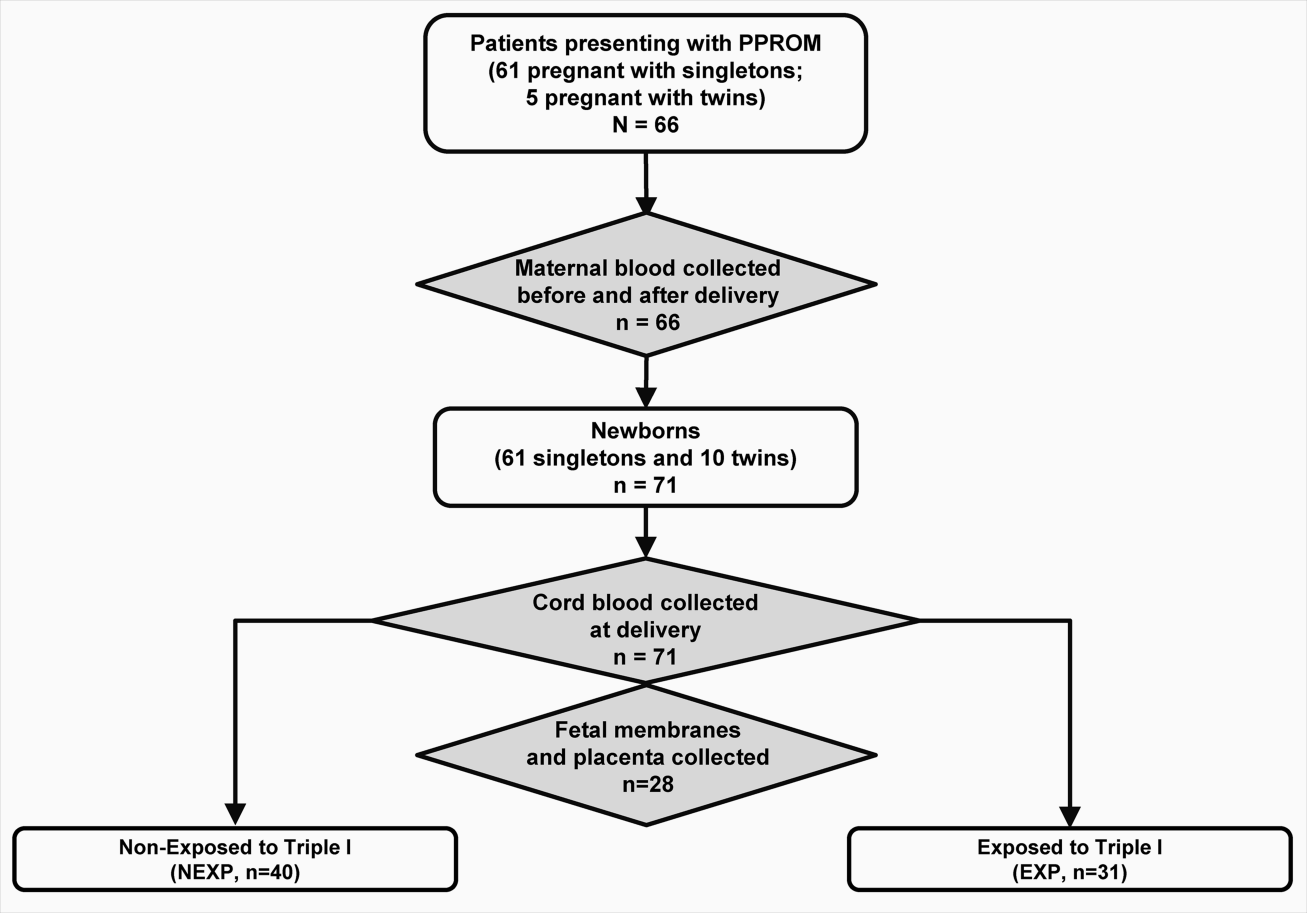
Flowchart of patients, newborns and biological samples analyzed part of this study

Bacterial DNA was identified above the level of the blank samples in 82% (54/66) of MB samples collected before birth and 85% (56/66) of the samples collected post-delivery. There was no difference in the bacterial DNA burden before vs. after delivery when data was normalized per MB volume (Wilcoxon test, p=0.085, Fig. 1A) or per ng total DNA (p=0.056, Fig. 1B). A significant decrease in bacterial load was identified after delivery when the normalization of the 16S RNA gene was done against the human housekeeping gene GPI (p=0.001, Fig. 1C). In 68% (45/66) of mothers, the circulating bacterial load decreased or remained unchanged after birth (Fig. 1D). Yet, in 21/66 (32%) pregnancies an increased level of bacterial DNA was measured (Fig. 1E). There was no difference in the proportion of EXP pregnancies between mothers who had an increase in circulating bacterial DNA burden postpartum (44%, 10/21) vs. those who had unchanged or decreased bacterial DNA postpartum (44%, 20/45, *p*=0.809). Lastly, we calculated the ratio in circulating bacterial DNA after:before delivery for each patient. We found no statistical difference in the peripartum change in bacterial DNA in MB between NEXP and EXP pregnancies (*p*=0.704, Fig. 1F).

**Figure 3:**
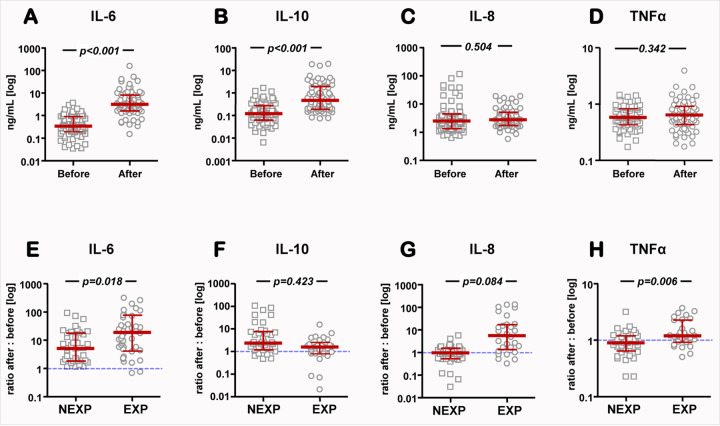
Scatterplots of maternal serum concentrations of interleukin-6 (IL-6, **A**), interleukin-8 (IL-8, **B**), interleukin-10 (IL-10, **C**) and tumor necrosis alpha (TNFα, **D**) before and 1-hour after delivery. Scatterplots comparing the maternal serum concentrations of IL-6 (**E**), IL-8, (**F**), IL-10, (**G**) and TNFα, (**G)** between non-exposed (NEXP, n=35) and exposed (EXP, n=31) mothers. Exposure status of the mother was determined by laboratory analysis of cord blood for haptoglobin and IL-6 as described in Methods. Mothers with twin gestations were considered EXP if at least one fetus was determined as EXP. **A-D:** The two-time points are compared by Wilcoxon signed rank test with Yates correction (n=66 mothers). **E-G:** The two groups are compared by Mann Whitney U-test. The group median (red thick horizontal line) and interquartile range are shown overlaid on each scatterplot.

**Figure 4:**
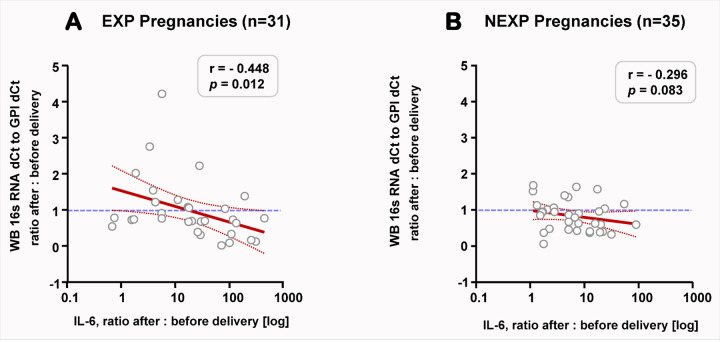
Relationship between the peripartum changes in maternal circulating bacterial DNA versus concentration of interleukin-6 (IL-6) as determined by ELISA (x-axis, log transformed) among exposed (EXP, **A**, n=31) and non-exposed (NEXP, **B,** n=35) mothers. Exposure status of the mother was determined by laboratory analysis of cord blood for haptoglobin and IL-6 as described in Methods. Mothers with twin gestations were considered EXP if at least one of their newborns was determined as EXP. The peripartum change was calculated from the ratio of the analyte after:before delivery. The blue dotted line represents a ratio of 1 (no change). The regression line is shown by the red thick line along with its confidence interval (dotted red lines).

Of the 10 cytokines measured in maternal serum, only IL-6, IL-8, IL-10 and TNFα measured within the limits of quantification in more than 50% of samples. Therefore, we restricted our analysis to these four cytokines. Compared to pre-delivery concentrations, maternal serum concentrations of IL-6 (Fig. 3A) and IL-10 (Fig. 3B) increased at 1h postpartum (Wilcoxon *p*<0.001 for both). No significant differences were recorded for IL-8 (Fig. 3C) or TNFα (Fig. 3D). However when the EXP and NEXP pregnancies were separated, the extent of postpartum increase in IL-6 (*p*=0.018, Fig. 3E) and TNFα (*p*=0.006, Fig. 3H) concentrations in the maternal circulation was significantly higher for EXP pregnancies. The significant increase in MB cytokine concentrations was maintained after correcting for delivery mode and GA at delivery in multivariable regression. Remarkably, we observed a significant inverse correlation between the change in IL-6 after delivery and the change in circulating bacterial DNA among pregnancies where an EXP neonate was identified (Fig. 4A, r= −0.448, *p*=0.012). This correlation was not significant among NEXP pregnancies (Fig. 4B, r= −0.296, *p*=0.083). In other words, in pregnancies complicated by PPROM and Triple I, the greater the increase in circulating maternal IL-6 after delivery, the greater the postpartum decrease in maternal circulating bacterial DNA. Relationships of circulating bacterial DNA with the other cytokines did not reach statistical significance for either group (not shown).

### Quantitative and qualitative relationships between bacterial footprints in maternal blood (MB) and reproductive tissues.

**Figure 5:**
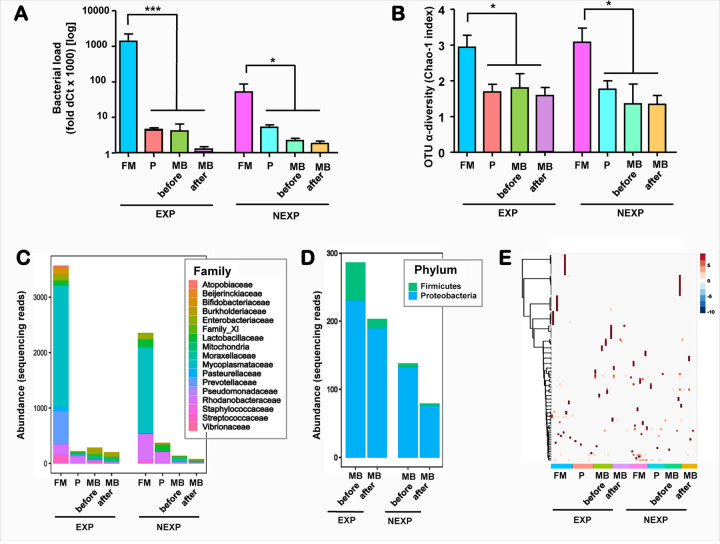
**A:** Quantitative relationships among bacterial load in fetal membranes, placental villous tissue and maternal blood (MB) before and after delivery in a subset of deliveries with matched samples (n=27 mothers and their 31 newborns). Bacterial load among different types of samples is appreciated by dCt for bacterial 16S RNA normalized to the human housekeeping gene glucose-6-phosphate isomerase (GPI) amplified in the same sample. **B:** Qualitative relationships in α-diversity appreciated by the Chao-1 index of operational taxonomic units (OTU, genus level) index among fetal membranes, **placental** villous tissue and MB before and after delivery. Exposure status of the newborns was determined by laboratory analysis of cord blood for haptoglobin and IL-6 as described in Methods. Of the 31 newborns 17 were identified as exposed (EXP) and 14 as non-exposed (NEXP). Data is shown as mean + SEM. Groups were compared using 2-way ANOVA after log transformation; Correlation between twins was adjusted using a Generalized Estimating Equation (GEE) model. *** p<0.001; * p<0.05; Qualitative relationships among samples appreciated by the relative representation of bacterial families **(C)** and phyla **(D)** among sequencing reads. **E:** Dendogram clustering of genus level operational taxonomic units (OTU) among samples from each delivery. The color intensity represents the number of reads in each sample.

To appreciate the relative amount of bacterial DNA captured in MB, we compared its level with that found in placental villous tissue and FM relative to the same housekeeping gene GPI. This analysis was limited to the cases with matched MB and delivery tissues (n=31). We found that FM harbored a higher bacterial biomass compared to placental villous tissue both among EXP (fold change bacterial DNA-to-GPI: FM 1.4 vs. Placenta 4.4 × 10^−3^, *p*<0.001) and NEXP pregnancies (FM 5.2 × 10^−2^ vs. Placenta 5.1 × 10^−3^, *p*=0.001) (Fig. 5A). There was no difference in the bacterial biomass by fetal EXP status in either FM or placenta. FM (but not placenta) had a higher bacterial burden than measured in MB both ante- and postpartum (p<0.001 for both).

Sequencing of the 16S amplicon libraries (n=110) identified 120 OTUs. As shown in Fig. 5B, FM harbored a higher OTU diversity than the other types of samples. These 120 OTUs belonged to 17 families with members the *Mycoplasmataceae* holding the top representation (Fig. 5C). Despite this, representatives of the *Mycoplasmataceae* family (or of the parent *Tenericutes* phylum) were not found in MB either pre- or post-delivery. Sequencing of the libraries from the MB samples identified only members of *Firmucutes* or *Proteobacteria* phyla with overall fewer reads in MB collected post-delivery (Fig. 5D). Fig 5E presents a heatmap of OTUs represented in each compartment. No taxa were systematically enriched in MB post-delivery by either LEfSe or by EdgeR. Sample by sample analysis identified three cases where the same OTU was identified both in MB and in the matched FM and four cases where a MB OTU had a match in placenta. However, the number of reads for these OTUs in each individual MB library was small (<10). Interestingly, the most abundant OTUs identified in MB had no match in either placenta or FM. All these OTUs belonged to the *Proteobacteria* phylum. Among them, OTUs of the genus *Escherichia-Shigella* and *Pseudomonas* were the most frequently identified both pre- and post-delivery.

[i]. Buhimschi CS, Bhandari V, Dulay AT, Nayeri UA, Abdel-Razeq SS, Pettker CM, Thung S, Zhao G, Han YW, Bizzarro M, Buhimschi IA. Proteomics mapping of cord blood identifies haptoglobin “switch-on” pattern as biomarker of early-onset neonatal sepsis in preterm newborns. PLoS One. 2011;6(10):e26111.

[ii]. Buhimschi CS, Jablonski KA, Rouse DJ, Varner MW, Reddy UM, Mercer BM, Leveno KJ, Wapner RJ, Sorokin Y, Thorp JM Jr, Ramin SM, Malone FD, Carpenter MW, O’Sullivan MJ, Peaceman AM, Saade GR, Dudley D, Caritis SN, Buhimschi IA; Eunice Kennedy Shriver National Institute of Child Health and Human Development Maternal-Fetal Medicine Units Network. Cord blood haptoglobin, cerebral palsy and death in infants of women at risk for preterm birth: a secondary analysis of a randomised controlled trial. EClinicalMedicine. 2019;9:11–18.

## Discussion

The present study continues to build on our previously reported concept of compartmentalization in pregnancies complicated by intra-amniotic infection and inflammation (Triple I).^24^ This model proposed that in the initial phases of the intra-amniotic infectious and inflammatory process, the maternal compartment is isolated from the fetus and its gestational sac through mechanisms set in place to protect the mother from infection. In PPROM, and especially in pregnancies with a long latency, the protective mechanisms become overpowered by the bacteria as they progressively gain access to the placental vessels from where the infectious process spreads. The first clinical sign of failure of the protective mechanisms are the occurrence of maternal leukocytosis accompanying Triple I.^27^ The compartmentalization model implies that in the setting of maternal systemic infections, the fetus is also protected because, in this reversed clinical circumstance, bacteria circulating in MB cannot reach the gestational sac. However, regardless of whether bacteria gain access to the placenta and to the maternal circulation through either a vaginal or hematogenous pathway, the current study provides molecular evidence that in 82% of cases microbes or microbial footprints are already circulating in MB after PPROM and prior to birth. However, as in our study, we cannot determine if bacteria were present in the MB prior to PPROM; that question requires further exploration.

Our study was inspired by the clinical observation that following birth, some women with long latent phases display transitory signs of sepsis, including hypotension, fever, chills, and shivering. To our knowledge, a systematic study investigating this clinical occurrence has not been reported in the literature. Several authors previously reported a frequency of maternal sepsis and bacteremia ranging from 1.5% to 14% in patients with either previable pregnancies, vaginal deliveries or Cesarean delivery after a minimum of 4 hours of ruptured membranes, respectively.^28,29,30^ Bogges et. al. reported that bacteremia was associated with presence of positive chorioamnionic cultures supporting our view and the currently presented data that fetal membranes may serve as a microbial reservoir from where the bacteria are seeded in maternal circulation. As shown in our analysis, in certain patients, inoculation of bacteria in the MB is heightened immediately after birth, possibly secondary to myometrial contraction and/or expulsion of the placenta. Yet, although overall the bacterial biomass circulating in MB before or immediately after delivery is small, compared to FM that harbor a much greater amount and diversity of bacteria, this phenomenon can trigger an immune response reflected by the observed selective cytokinemia.

Clearance of microorganisms from blood has been a subject of longstanding debate and substantial research. Bacterial bloodstream clearance is a very rapid process controlled through various mechanisms, including opsonophagocytosis and oxycytosis.^31,32^ In opsonophagocytosis bacteria are engulfed by phagocytic cell (neutrophils and monocytes in blood and macrophages in tissues) where they are opsonized with antibodies and/or complement proteins. While opsonophagocytosis effectively clears bacteria from infected tissues, its role in removing bloodstream bacteria has been challenged and a role for erythrocytes has been proposed: bacteria adheres to erythrocytes by electric charge attraction (triboelectric effect) and is killed by oxygen free radicals released by oxyhemoglobin in a phenomenon called oxycytosis. Because we isolated bacterial DNA from an aliquot of whole blood, our results would have captured bacteria and bacterial fragments present both inside and outside blood cells. Our data could not account for the bacteria that has entered the bloodstream but then removed by the spleen and liver within 1 hour after delivery.

Aside from the oral cavity, the gut represents another bacterial reservoir from which bacteria are frequently translocated to the blood stream part of normal human physiology or in pathological conditions.^33^ Particular to pregnancy, the amniotic cavity and reproductive tissues can also serve as bacterial reservoirs should they become seeded by bacteria. Our current study suggests that of all reproductive tissues FM harbors the richest microbiome in the context of PPROM. The fact that the FM bacteria do not gain access to the maternal bloodstream or are rapidly neutralized in most patients was unexpected.

In this study, we limited our enrollment to patients with PPROM. Therefore, we were not able to assess if the circulating bacterial biomass is decreased or increased compared to patients with intact membranes. This comparison would be difficult since all PPROM patients ultimately receive broadspectrum antibiotics, which by themselves could play a role, whereas patients with intact membranes are only given a similar antibiotic regimen if there is a suspicion of infection.

Although blood was conventionally thought to be sterile, emerging studies are increasingly identifying a low biomass blood microbiome reflective of both health and disease.^34,35^ The chief controversy of these studies is the susceptibility of any low biomass result to environmental contamination during sample collection, handling of laboratory materials, or during downstream procedures of DNA extraction and amplification. Additionally, questions have been raised about the clinical relevance of blood microbiota based on culture-independent methods in the absence of the demonstration of microbial viability. Thus far, there is no support for a healthy core blood microbiome.^36^ Rather, data from several studies agree on transient and sporadic translocations of commensal bacteria into the bloodstream, from where they are rapidly cleared. Therefore the notion of disease may actually coincide with the insufficiency of the clearance mechanisms in the blood relative to the size of the inoculum.^34^ The current study concurs with the above assertion. Even in the context of PPROM and Triple I, where the FM have a high bacterial burden, the clearance mechanisms are effective in decreasing the amount of bacterial DNA in correlation with the increase in circulating cytokines. Given the rarity of sepsis in PPROM patients who are all receiving antibiotics by standard of care, we were unable to capture patients who developed sepsis, and this is a limitation of the current study.

In this study we analyzed presence of cultivable and uncultivable bacteria immediately after PPROM and following expulsion of the fetus and its placenta. To our knowledge, for the first time, we employed culture independent techniques to identify presence of bacteria in the MB of mothers presenting with PPROM. The increased sensitivity of the molecular techniques was obvious through the observation that 82% and 85% of MB samples tested positive for bacterial DNA before and after birth, respectively. Quantification of the bacterial DNA load was novel considering that, to our knowledge, in all previous studies, the presence of bacteremia after placental separation was employed only with qualitative culture-dependent methods. Short lived bacteremia after vaginal or cesarean delivery was previously reported.^30^ Interestingly, in this study, 68% of our PPROM patients registered a significant decrease or no change in the MB bacterial load after birth. We opine that this could be the group of patients where delivery of the FM eliminated the source of MB bacterial seeding or the clearance mechanisms were rapidly activated. On the other hand, in some patients, the observed decrease in the bacterial biomass following delivery of the placenta was associated with a robust inflammatory response, especially in MB for EXP pregnancies with higher circulating levels of IL-6. Therefore, the decrease in the MB bacterial mass and activation of the inflammatory response, which in turn augments the opsonophagocytic mechanisms are also likely linked.^37^

Unique to the current analysis is the observation that in the immediate post-partum period bacterial DNA in MB increased in 32% of PPROM women. An overwhelming release of amnio-chorion bacteria in the maternal circulation, an inefficient innate immune response and/or bacterial clearance mechanisms may explain our observations. Interestingly, our data revealed that the post-delivery the cytokine response was selective with just IL-6 and IL-10 being upregulated. This is not surprising, considering that previously published work reported that the context of the immune response, including the type of pathogen involved, may impact on the involved regulatory mechanisms.^38^ Yet, IL-6 and IL-10 are cytokines with significant anti-inflammatory properties that plays key functions in limiting host immune response to pathogens, thereby preventing damage to the host.^38,39^

Analysis of the cord blood identified that almost half of the newborns were EXP in-utero to a bacterial pro-inflammatory stimulus. A longer latency period and a lower GA at PPROM were identified as factors that played a significant role. This was not surprising because longer latencies increase the risk of bacterial ascension and multiplication in the gestational sac and favors microbial access to the fetal and maternal blood compartments at lower GAs.^40^ In our study, at birth, 90% of the bacterial EXP neonates displayed a cord blood Hp switch-on pattern and had higher IL-6 levels compared to NEXP newborns. In the same clinical scenario, MB WBC count and IL-6 levels were significantly elevated with a significant inverse correlation between MB IL-6 levels and the size of the bacterial DNA load. Taken together, these observations suggest that fetal exposure to Triple I stimulates a maternal cytokine response, which in turn decreases the size of any bacterial DNA load. While this connection is counterintuitive in the context of the compartmentalization it supports the notion of an active feto-maternal immune communication.

Sequencing of the 16S amplicon libraries identified 120 OTUs, and FM harbored a higher OTU diversity than the other types of samples, with no difference between EXP and NEXP pregnancies. These 120 OTUs belonged to 17 families with members the *Mycoplasmataceae* holding the top representation. Interestingly, representatives of the *Mycoplasmataceae* family (or of the parent *Tenericutes* phylum) were not found in MB either pre- or post-delivery. Laboratory contamination and blood cultivation in the absence of sequencing may explain why in previous studies, but not ours, Mycoplasma was identified shortly after vaginal delivery.^30^ Sequencing of the libraries from the MB samples identified only members of *Firmucutes* (dominant part of the microbiota in the human gut) or *Proteobacteria* phyla (the most important phylum of Gram-negative bacteria that has multiple habitats including oral cavity, gastrointestinal tract and vagina).^41,42^ Interestingly, the most abundant MB OTUs (*Proteobacteria* phylum) had no match in either placenta or FM. Among them, OTUs of the genus *Escherichia-Shigella* (Gram-negative genus of bacteria genetically related to Escherichia coli) and *Pseudomonas* were the most frequently identified both pre- and post-delivery. If our interpretation is correct, the initial hypothesis that following birth bacteria trapped in placenta or FM translocate to MB is rejected. Our sequencing data suggests that the MB microbiome has most of its origin in the bowel. Based on the evidence that some *Shigella* spp. and *Escherichia coli* strains contain a virulence plasmid of ~220 kb that favors entry into epithelial cells and dissemination from cell to cell, we believe there is biological plausibility for our assumption.^43^

## Conclusions

In PPROM, bacterial DNA in the maternal bloodstream is rapidly eliminated, coinciding with a significant increase in circulating cytokines. While fetal membranes harbor a substantial microbiome regardless of the presence of Triple I, these bacteria do not seem to translocate to maternal blood in significant amounts under current clinical conditions. Further studies are needed to elucidate the pathogen-host interactions that allow microbial persistence in maternal circulation in the setting of PPROM.

## Methods

### Study design and patients.

Using a prospective observational study design, we enrolled 66 consecutive pregnant patients who were admitted on the antepartum service of our academic university hospital for management of PPROM. All subjects were recruited at The Ohio State University Wexner Medical Center and provided written informed consent. Experimental protocol, data collection, and consent forms were approved by the Institutional Review Boards of The Ohio State University and Nationwide Children’s Hospital. All procedures were performed in accordance with the relevant guidelines and regulations.

Subjects were eligible for enrollment if older than 18 years of age, pregnant between 23^0/7^ to 33^6/7^ weeks gestational age and at high risk of preterm birth secondary to PPROM. Exclusion criteria were anticipated delivery within 2 hours, need for close medical supervision (e.g. cardiac and renal disease, congestive heart failure, history of asthma), maternal viral infection (HIV, hepatitis B or C), known active infections or antibiotic use 7 days prior to admission, need for emergent delivery (e.g. cord prolapse, abruption) and known fetal malformations.

GA was determined based on last menstrual period confirmed by an ultrasound examination at 18–20 weeks GA.^44^ Preterm labor was defined as documented cervical effacement and/or dilatation in the presence of regular uterine contractions.^45^ PPROM was diagnosed by vaginal AF “pooling,” and/or “ferning” and/or confirmed by an amniocentesis-dye positive test.^46^

Following admission, expectant management, antibiotics [(ampicillin 2 g IV every 6 hours and a single oral dose of azithromycin 1 g for 48 h), followed by oral amoxicillin (250 mg every 8 hours) to prolong pregnancy], steroids and magnesium sulfate for neuroprotection were considered for all patients based on recognized American College of Obstetrics and Gynecology recommendations and clinical circumstances.^47^ A vaginal–rectal swab for group B streptococcus (GBS) culture was obtained at the time of admission and GBS prophylaxis administered during labor as indicated. When the diagnosis of Triple I was suspected due to fever and associated clinical risk factors or confirmed by positive amniocentesis results, delivery was indicated independent of our research protocol. Additionally, for these cases broad-spectrum antibiotics were started immediately (ampicillin 2 g IV every 6 hours and gentamicin 1.5 mg/kg every 8 hours, or other broad-spectrum regimens in patients with penicillin allergies).^48^

### Biological samples.

Maternal blood (MB) was retrieved via sterile antecubital venipuncture prior to birth and within 1-hour post-partum (PP). An alcohol swab was used to prepare the skin area before venipuncture. The blood was collected in serum and plasma citrate tubes (BD Vacutainer, Franklin Lakes, NJ) and transported to the laboratory. An aliquot of whole blood from the plasma tube was first saved for bacterial DNA isolation. The remainder blood volume was spun at 1,500 g for 15 min and plasma and serum aliquoted for cytokine measurements.

Cord blood was collected at delivery from all newborns of mothers enrolled in this study. A total of n=71 cord blood sample were available for analysis: 61 samples from singleton newborns and 10 samples from the 5 twin pairs. After delivery of the placenta, a loop of the umbilical cord was placed on a sterile field and cord blood was collected by sterile puncture of the umbilical vein. The cord blood was processed as described above for MB.

In a subset of deliveries (n=31 newborns: 23 singletons and 4 pairs of twins), a biopsy from FM (full thickness amniochorion) and from the villous tissue in center of the placental disk were retrieved in strict sterile fashion within minutes from delivery. Collection of delivery tissues was contingent on availability of research staff at delivery. Following dissection of the decidua basalis, the villous trophoblast tissue was frozen immediately in liquid nitrogen. The fetal membrane biopsy was rinsed briefly in sterile saline to remove any AF and adherent blood prior to freezing in sterile DNA-free tubes.

All blood, serum, plasma and tissue samples were stored at −80°C until further isolations and analyses were performed. All materials were purchased as DNA and RNA-free and blank samples were processed in parallel to assess for possible environmental contamination.

### DNA extraction and measurement of bacterial biomass.

The laboratorypersonnel did not have access to the clinical variables prior to analysis of the data. DNA was isolated with the bead-beating method and cleaned up with QIAamp DNA mini-kit (Qiagen, Germantown, MD). DNA quantification and quality check was performed with Thermo Scientific NanoDrop 2000/2000c Spectrophotometer (Thermo Fisher Scientific, Waltham MA). All procedures were carried out under strict sterile conditions.

The 16S rRNA gene was amplified using a protocol used in a prior study.^49^ The primers and probes (Integrated DNA Technologies, Coralville Iowa) were as follows: forward primer, 5’-TCC TAC GGG AGG CAG CAG T-3’ (Tm, 60.8 °C), reverse primer, 5’-GGA CTA CCA GGG TAT CTA ATC CTG TT-3’(Tm, 57.2 °C), and 16S universal probe, 5’-/56-FAM/CGT ATT ACC GCG GCT GGC AC/36-TAMSp/-3 (Tm, 65.6 °C).^50^ Glucose-6-phosphate isomerase (GPI) was amplified using TaqMan Gene Expression Assay (Hs03882242_s1, Applied Biosystems, Foster City, CA) and used as housekeeping gene based on its qualification of “exceptionally uniform gene”.^51^ Additionally, for absolute determination of bacterial biomass, a standard curve was generated from a bacterial stock of *Porphyromonas gingivalis* (ATCC, Manassas, VA). Each 20 μl reaction consisted of 1 μl DNA, 1 μl of 16S primers/probe set (20x) or TaqMan Gene Expression Assay, 10 μl TaqMan Fast Advanced Master Mix (Applied Biosystems, Foster City, CA), and 8 μl of nuclease free water. Amplifications were performed in the StepOnePlus Real-Time PCR System (Applied Biosystems, Foster City, CA). Whole blood spiked with known bacterial cfu was used as technical control for recovery of bacterial DNA. Environmental samples containing sterile water added to the same type of tubes as those used for blood collection were processed simultaneously in each batch and all failed to amplify.

### Sequencing of 16s RNA genes and microbial community analysis.

Genomic DNA was extracted and the 16S rRNA amplified using V4 primers. 16S amplicon multiplex libraries were sequenced on Illumina platform at the Sequencing Facility of the Argonne National Laboratory and mapped to the SILVA database (https://www.arb-silva.de/) using QIIME2. An operational taxonomic units (OTU) table was generated which contained the OTUs identified in each sample along with the number of sequence reads and the taxonomic classification of each OTU. The classification was further collapsed at either genera, family, order or phylum level for subsequent analyses using the MicrobialAnalyst web-based platform (https://www.microbiomeanalyst.ca/).^52^ Because blood samples were expected to have a low number of reads, we thought more important to be inclusive of low abundance taxa even if these could represent sequencing errors, therefore the default R code was modified to exclude the abundance filters. The Chao-1 index (measure of α-diversity) was used to estimate OTU richness in different types of samples. Enrichment of taxa between groups was further analyzed by LEfSe (linear discriminant analysis effect size) and EdgeR methods built within the platform. These computational algorithms designed primarily for highly multidimensional data, employ a combination of statistical tests to extract features (e.g. OTU) that show significant differences in abundance (enrichment) across samples while accounting for the effect size of the differences.

### Maternal circulating cytokines.

Maternal serum before and after delivery was analyzed by electrochemiluminescence-based immunoassay for a panel of 10 pro- and anti-inflammatory cytokines that included IFN-γ, TNF-α, IL-1β, IL-2, IL-4, IL-6, IL-8, IL-10, IL12p70, and IL-13 (V-PLEX Proinflammatory Panel 1 Human, Meso Scale Discovery, Rockville, MD). Assays were performed according to the manufacturer’s instructions with minimal modifications. Briefly, wells of the 96-well plates contained 50 μL of 1:2 diluted serum sample or high, medium, and low quality control samples supplied by the manufacturer. Plates were incubated at room temperature for 2.5 hours with continuous shaking then washed three times with 1× Wash Buffer. Following addition of SULFO-TAG Detection Antibody Cocktail, plates were incubated for 2 hours with shaking at room temperature, washed, and following addition of 150 μL Read Buffer per well, plates were scanned with a SECTOR Imager 6000 Reader (Meso Scale Discovery). Raw signals generated by the instrument were analyzed with Discovery Workbench 4.0 Software (Meso Scale Discovery). All values are reported in units of pg/mL. Samples were measured in duplicate and averaged for analysis. The coefficient of variation for inter- and intra-assay variability was <7% for all assays.

### Assessment of antenatal exposure to inflammation based on cord blood biomarkers.

The combination of cord blood haptoglobin (Hp) switch status and cord blood IL-6 was used to determine whether the fetus has been exposed (EXP) or non-exposed (NEXP) to Triple I and was able to properly respond by upregulating its endogenous Hp production. Hp concentration was measured using 1:150 and 1:5,000 dilutions in blocking buffer (1% non-fat dry milk). A 7-point standard curve (250–3.9 ng/mL) was prepared from mixed phenotype Hp (Hp1–2, Sigma). The minimal detectable concentration for Hp was 1.5 ng/mL. As previously described, western blots were performed on all samples measuring ≥2,000 ng/mL in Hp ELISA. SDS-PAGE Gels (10–20%, Invitrogen) were loaded with equal amounts of cord plasma protein (2 μg/lane) mixed 1:2 with reducing sample buffer (Bio-Rad) and boiled for 5 min.^25,26^ After electrophoretic transfer, nitrocellulose membranes (Bio-Rad) were blocked with 5% milk and then incubated overnight at 4°C rabbit anti-Hp polyclonal antibody (1:3000, Sigma). Detection was performed using biotinylated goat anti-rabbit secondary antibody (1:5000, Jackson Immunoresearch) followed by streptavidin-linked horseradish peroxidase, (1:8000, Amersham Biosystems), chemiluminescence (ECL for low sensitivity exposure and ECL Plus for high sensitivity, Amersham) each with 1-minute timed exposure to film (Kodak Biomax). A detectable Hp β-chain (~42 kDa) on a western blot was indicative of a switch-on pattern in Hp expression. Cord blood IL-6 was measured with a commercial high sensitivity ELISA assay (R&D Systems).

Newborns with cord blood IL-6 <100 pg/mL and switch-off Hp pattern (anhaptoglobinemic) were considered “non-exposed to Triple I” (NEXP) while haptoglobinemic newborns (Hp switch-on pattern) were considered “exposed to Triple I” (EXP) irrespective of the cord blood IL-6 concentration as previously described.^25,26^

### Additional statistical analyses.

Normality testing was performed using the Shapiro-Wilk test. Bacterial load data was analyzed after logarithmic transformation. Comparisons employed Mann-Whitney U-tests or Wilcoxon Signed Rank test (comparing signals before and after delivery), chi square test or Fisher’s exact tests (comparing proportions). Correlation between twins was adjusted using a Generalized Estimating Equation (GEE) model. A p-value of ≤0.05 was considered statistically significant. SigmaPlot (ver.14 Systat Software) and SPSS Advanced Statistics (ver. 26 IBM) were used as aids for statistical analyses.

## Supplementary Files

This is a list of supplementary files associated with this preprint. Click to download.

- Table1PPROMbacteriabloodstream081225.docx
- Table2PPROMbacteriabloodstream081225.docx

## Figures and Tables

**Figure 2:**
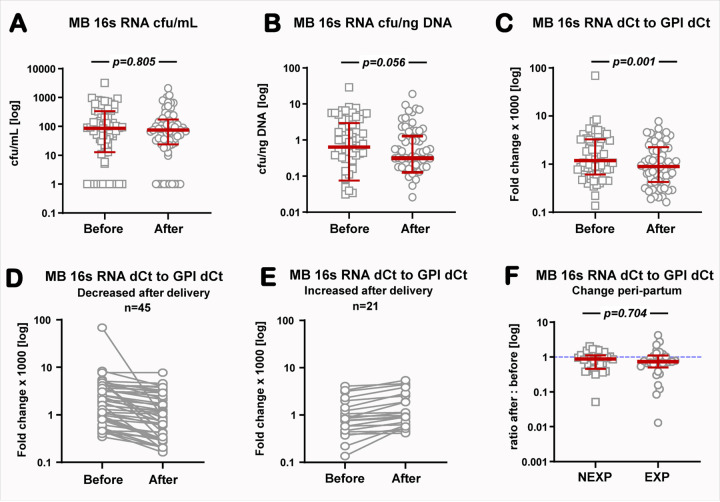
Bacterial load in maternal blood (MB) before (n=66) and after birth (n=66) measured by 16S rRNA PCR and normalized to the volume of blood **(A)**, total DNA **(B)** or the human housekeeping gene glucose-6-phosphate isomerase (GPI) amplified in the same sample **(C).** Level of change in MB bacterial load normalized to GPI for patients where a decrease (**D**, n=45) or an increase in bacterial load was observed (**E**, n=21) after delivery. **F:** change in MB bacterial load peri-partum expressed as a ratio after:before delivery for mothers with fetuses non-exposed (NEXP, n=35) or exposed (EXP, n=31) to Triple I. Of the five twin pregnancies included in the study, two pregnancies were classified as EXP based on one of the fetuses presenting the cord blood biomarkers of antenatal exposure to infection/inflammation (Triple I). **A-C & F**: Scatterplots with group median (red thick horizontal line) and interquartile range. Groups are compared with Wilcoxon signed rank test with Yates correction. **D&E**: Symbols represent levels before and after birth for each patient connected by a line.

## Data Availability

The anonymized dataset generated and analyzed during the current study is available from the Zenodo repository at: https://doi.org/10.5281/zenodo.16898408. Patient level clinical data is not openly available due to privacy concerns but is available from the corresponding author upon reasonable request. The raw and processed amplicon sequencing data are available in the Gene Expression Omnibus (GEO) database at the accession number GSE306804.
